# The crystal structure of TlMgCl_3_ from 290 K to 725 K

**DOI:** 10.1107/S2056989020013201

**Published:** 2020-10-06

**Authors:** Drew R. Onken, Didier Perrodin, Sven C. Vogel, Edith D. Bourret, Federico Moretti

**Affiliations:** a Lawrence Berkeley National Laboratory, Berkeley, CA 94720, USA; b Los Alamos National Laboratory, Los Alamos, NM 87545, USA

**Keywords:** TlMgCl_3_, scintillator, crystal structure, high-temperature neutron diffraction

## Abstract

The title compound, thallium magnesium trichloride, has been identified as a scintillator with both moderate gamma-stopping power and moderate light yield. Knowledge of its crystal structure is needed for further development. This work determines the crystal structure of TlMgCl_3_ to be hexa­gonal *P*6_3_/*mmc* (No. 194) and isostructural with RbMgCl_3_, contrary to previously reported data. This structure was obtained by single-crystal X-ray diffraction and was further confirmed by neutron diffraction measurements. Extending neutron diffraction measurements to high temperature, the data show that TlMgCl_3_ maintains this crystal structure from 290 K up through 725 K, approaching the melting point of 770 K. Anisotropic thermal expansion coefficients increase over this temperature range, from 31 to 38 × 10^−6^ K^−1^ along the *a* axis and from 19 to 34 × 10^−6^ K^−1^ along the *c* axis.

## Abstract 2

The title compound, thallium magnesium trichloride, has been identified as a scintillator with both moderate gamma-stopping power and moderate light yield. Knowledge of its crystal structure is needed for further development. This work determines the crystal structure of TlMgCl_3_ to be hexa­gonal *P*6_3_/*mmc* (No. 194) and isostructural with RbMgCl_3_, contrary to previously reported data. Extending neutron diffraction measurements to high temperature, the data show that TlMgCl_3_ maintains this crystal structure from 290 K up through 725 K, approaching the melting point of 770 K.

## Chemical context   

In the ongoing search for inorganic scintillators with high gamma-stopping power, TlMgCl_3_ has been identified. As a result of the presence of thallium, TlMgCl_3_ has a high effective atomic number, *Z*_eff_ = 67 [calculation methodology (Derenzo & Choong, 2009 ▸) in the supporting information], and a moderate density, ρ = 4.47 g cm^−3^ (determined in this work). A pair of initial crystal growths of TlMgCl_3_ have been conducted to assess the scintillation properties: Fujimoto *et al.* (2016 ▸) measured 46,000 ph MeV^−1^ light yield with 5% energy resolution at 662 keV, and Hawrami *et al.* (2017 ▸) measured 30,600 ph MeV^−1^ light yield with 3.7% energy resolution at 662 keV.

To develop this compound further, a precise determination of the crystal structure is necessary. This will enable first-principles calculations of the electronic configuration and may be useful in assessing challenges that arise during synthesis (*e.g*. from thermal stresses). This work reports the crystal structure of TlMgCl_3_ between 290 K and 725 K, approaching the melting point of 770 K. Previous work on TlMgCl_3_ by Beznosikov (1978 ▸) used powder diffraction to report the space group at room temperature as ortho­rhom­bic (*a* = 6.54, *b* = 9.22, *c* = 6.99 Å). However, despite using the same synthesis procedure, the structure reported by Beznosikov does not fit the diffraction data reported herein. Arai *et al.* (2020 ▸) published diffraction data but did not provide information on the crystal structure.

## Structural commentary   

**Figure 1:**
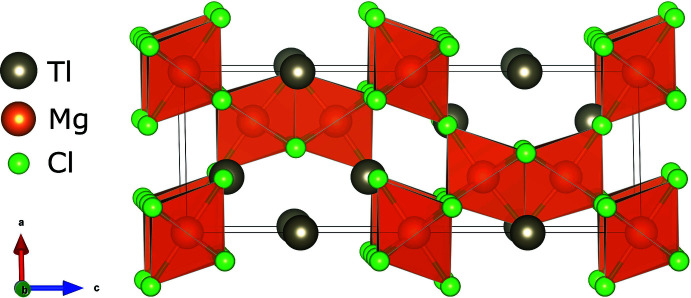
The unit cell of TlMgCl_3_, with the MgCl_6_ octa­hedra shown in polyhedral representation.

Single crystal X-ray diffraction (SC-XRD) determined TlMgCl_3_ to have a hexa­gonal structure (space group *P*6_3_/*mmc*, No. 194) with lattice parameters *a* = 7.0228 (4), *c* = 17.4934 (15) Å at 290 K. Fig. 1 ▸ visualizes the unit cell, which shows a three-dimensional corner- and face-sharing framework of six-coordinated Mg atoms encapsulating the 12-coordinated Tl atoms. There are six formula units in the unit cell. There are two thallium, two magnesium and two chlorine atoms in the asymmetric unit of TlMgCl_3_, with site symmetries of 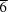 *m*2 and 3*m*; 3*m* and 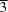 *m*; *mm*2 and *m*, respectively; key bond distances and angles are listed in Table 1 ▸. Pairs of Mg2-centered octa­hedra share faces (*via* 3 × Cl1) and these octa­hedral pairs share corners (*via* Cl2) with the Mg1 octa­hedra to generate an *ABACBC* hexa­gonal stacking sequence of the chloride ions in the *c*-axis direction with the thallium cations occupying the vacant 12-coordinate sites. The coordination polyhedra of the chloride ions are distorted ClMg_2_Tl_4_ octa­hedra with the Mg^2+^ ions in a *cis* disposition for Cl1 and a *trans* disposition for Cl2. The title compound is isostructural with RbMgCl_3_ as reported by Devaney *et al.* (1981 ▸) and RbMnCl_3_ as reported by Goodyear *et al.* (1977 ▸), who describe the structure in more detail. This structure is more complex than that of CsMgCl_3_ (McPherson *et al.*, 1970 ▸), which also has space group *P*6_3_/*mmc* but only requires two formula units per unit cell and has an *AB* hexa­gonal stacking sequence of the chloride ions in the *c*-axis direction.

**Figure 2:**
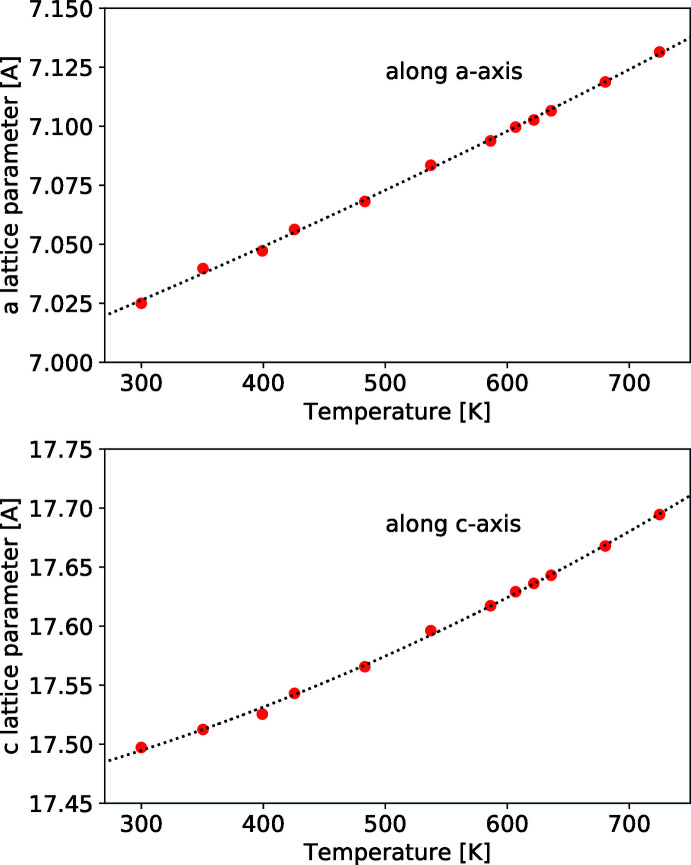
The hexa­gonal lattice parameters of TlMgCl_3_ as a function of temperature, from neutron diffraction data. Vertical error bars from Rietveld fitting are within the size of the symbols and are omitted. The dashed lines are second-order polynomial fits to the data.

**Figure 3:**
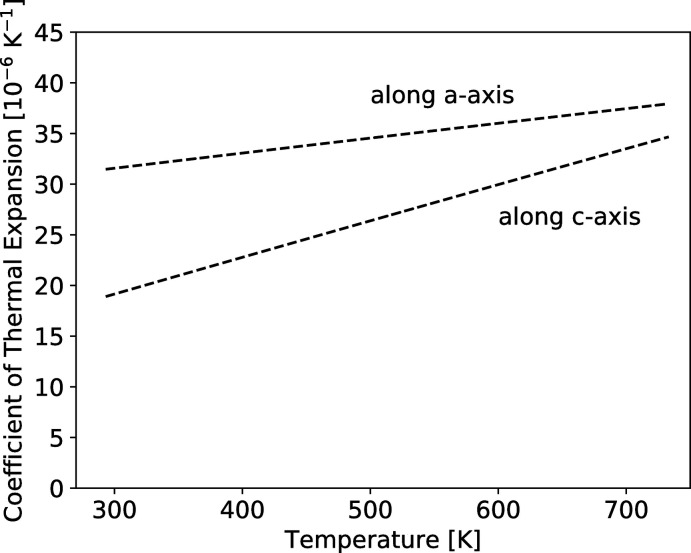
Thermal expansion coefficients as a function of temperature, calculated from the second-order polynomial fit of the lattice parameters in Fig. 2 ▸.

Neutron diffraction (ND) conducted on powder samples produced diffraction patterns that were in agreement with the crystal structure determined by SC-XRD. Neutron diffraction was conducted at temperatures ranging from 300 K to 725 K. TlMgCl_3_ maintains the same *P*6_3_/*mmc* crystal structure over this measured temperature range (see supporting information for more details on the powder ND data and fits). Fig. 2 ▸ shows the lattice parameters as a function of temperature. From these data, the thermal expansion along each axis is calculated (Fig. 3 ▸). The thermal expansion is greater along the *a* axis than the *c* axis. Besides the anisotropy in the lattice parameters, the atomic positions did not vary significantly with temperature, and therefore the bond lengths change with temperature as dictated by the lattice parameters alone.

## Synthesis and crystallization   

Crystals of TlMgCl_3_ were grown from the melt using the vertical Bridgman method. High purity beads of TlCl and MgCl_2_ were combined in a stoichiometric ratio and sealed in a quartz ampoule under vacuum (10^−6^ Torr). The crystal was grown with a translation speed of 0.5 mm h^−1^ and was cooled over 72 h. To protect the moisture-sensitive reactants and products, all preparations before and after synthesis were conducted inside an argon-filled glove box.

## Refinement   

SC-XRD was conducted on a Bruker Kappa APEXII CCD diffractometer. The crystal was protected from moisture by oil during mounting and by an Oxford dry nitro­gen gas cryostream system during data collection at 290 K. Crystal data, data collection and structure refinement details are summarized in Table 2 ▸.

Powder high-temperature ND measurements were obtained using the high-pressure preferred orientation (HIPPO) neutron diffractometer at the short-pulsed spallation neutron source of the Lujan Neutron Scattering Center at Los Alamos National Laboratory (Wenk *et al.*, 2003 ▸; Vogel *et al.*, 2004 ▸). Powder samples were sealed under argon in vanadium tubes to protect from moisture during data collection. Time-of-flight data were collected with HIPPO detector panels of ^3^He detector tubes arranged on five rings with nominal diffraction angles of 2θ = 39, 60, 90, 120, and 144°. Count times were 90 minutes per dwell time. ND data were analyzed for all five rings simultaneously using the Rietveld method implemented in the *GSAS* code (Larson & Von Dreele, 2004 ▸) and automated by scripts through gsaslanguage (Vogel, 2011 ▸). To yield reliable absolute lattice parameters, the DIFC instrument calibration parameters were fitted for the room-temperature data using the lattice parameters from SC-XRD and were kept constant for the rest of the ND data at higher temperatures. For more details on the data collection and refinement of these neutron diffraction data, see Onken *et al.* (2018 ▸).

The thermal expansion tensor was generated using a quadratic fit to the lattice parameters (*R* ^2^ = 0.999), using the *Thermal Expansion Visualization* (TEV) program (Langreiter & Kahlenberg, 2015 ▸).

## Supplementary Material

Crystal structure: contains datablock(s) I, global. DOI: 10.1107/S2056989020013201/hb7945sup1.cif

Structure factors: contains datablock(s) I. DOI: 10.1107/S2056989020013201/hb7945Isup2.hkl

Supporting information includes additional details, plots, and tables describing the high-temperature neutron diffraction data and refinements. DOI: 10.1107/S2056989020013201/hb7945sup3.odt

CCDC reference: 2034695

Additional supporting information:  crystallographic information; 3D view; checkCIF report

## Figures and Tables

**Table 1:** Selected geometric parameters (Å, °)

Tl1—Cl1	3.5126 (2)	Tl2—Cl2^iv^	3.622 (5)
Tl1—Cl2^i^	3.576 (5)	Mg1—Cl2	2.448 (6)
Tl2—Cl1^ii^	3.510 (3)	Mg1—Cl1	2.499 (6)
Tl2—Cl2^iii^	3.5146 (3)	Mg2—Cl2	2.476 (4)
			
Mg1^v^—Cl1—Mg1	78.5 (3)	Mg1—Cl2—Mg2	178.8 (3)

Symmetry codes: (i) 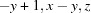; (ii) 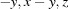; (iii) 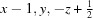; (iv) 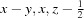; (v) 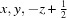.

**Table 2:** Experimental details

Crystal data	
Chemical formula	TlMgCl_3_
*M*_r_	335.03
Crystal system, space group	Hexagonal, *P*6_3_/*m* *m* *c*
Temperature (K)	290
*a*, *c* (Å)	7.0228 (4), 17.4934 (15)
*V* (Å^3^)	747.18 (11)
*Z*	6
Radiation type	Mo *K*α
μ (mm^−1^)	33.97
Crystal size (mm)	0.10 × 0.10 × 0.10

Data collection	
Diffractometer	Bruker Kappa APEXII CCD
Absorption correction	Multi-scan (*SADABS*; Bruker, 2004 ▸)
*T*_min_, *T*_max_	0.578, 0.746
No. of measured, independent and observed [*I* > 2σ(*I*)] reflections	4109, 489, 387
*R*_int_	0.047
(sin θ/λ)_max_ (Å^−1^)	0.718

Refinement	
*R*[*F* ^2^ > 2σ(*F* ^2^)], *wR*(*F* ^2^), *S*	0.047, 0.113, 1.42
No. of reflections	489
No. of parameters	21
Δρ_max_, Δρ_min_ (e Å^−3^)	1.88, −2.11

Computer programs: *APEX2* and *SAINT* (Bruker, 2004 ▸), *SHELXT2014/4* (Sheldrick, 2015*a* ▸), *SHELXL2018/3* (Sheldrick, 2015*b* ▸) and *VESTA* (Momma & Izumi, 2011 ▸).

## supplementary crystallographic information

### Crystal data

**Table:**

TlMgCl_3_	*D*_x_ = 4.467 Mg m^−^^3^
*M**_r_* = 335.03	Mo *K*α radiation, λ = 0.71073 Å
Hexagonal, *P*6_3_/*m**m**c*	Cell parameters from 1053 reflections
*a* = 7.0228 (4) Å	θ = 3.6–28.2°
*c* = 17.4934 (15) Å	µ = 33.97 mm^−^^1^
*V* = 747.18 (11) Å^3^	*T* = 290 K
*Z* = 6	Block, colorless
*F*(000) = 864	0.10 × 0.10 × 0.10 mm

### Data collection

**Table:**

Bruker Kappa APEXII CCD diffractometer	387 reflections with *I* > 2σ(*I*)
ω scans	*R*_int_ = 0.047
Absorption correction: multi-scan (SADABS; Bruker, 2004)	θ_max_ = 30.7°, θ_min_ = 2.3°
*T*_min_ = 0.578, *T*_max_ = 0.746	*h* = −8→9
4109 measured reflections	*k* = −8→8
489 independent reflections	*l* = −23→25

### Refinement

**Table:**

Refinement on *F*^2^	0 restraints
Least-squares matrix: full	Primary atom site location: dual
*R*[*F*^2^ > 2σ(*F*^2^)] = 0.047	*w* = 1/[σ^2^(*F*_o_^2^) + 23.1798*P*] where *P* = (*F*_o_^2^ + 2*F*_c_^2^)/3
*wR*(*F*^2^) = 0.113	(Δ/σ)_max_ < 0.001
*S* = 1.42	Δρ_max_ = 1.88 e Å^−^^3^
489 reflections	Δρ_min_ = −2.11 e Å^−^^3^
21 parameters	

### Special details

**Table:**

Geometry. All esds (except the esd in the dihedral angle between two l.s. planes) are estimated using the full covariance matrix. The cell esds are taken into account individually in the estimation of esds in distances, angles and torsion angles; correlations between esds in cell parameters are only used when they are defined by crystal symmetry. An approximate (isotropic) treatment of cell esds is used for estimating esds involving l.s. planes.

### Fractional atomic coordinates and isotropic or equivalent isotropic displacement parameters (Å^2^)

**Table:**

	*x*	*y*	*z*	*U*_iso_*/*U*_eq_	
Tl1	0.000000	0.000000	0.250000	0.0375 (5)	
Tl2	0.333333	0.666667	0.09002 (7)	0.0395 (4)	
Mg1	0.666667	0.333333	0.3404 (4)	0.0115 (14)	
Mg2	1.000000	1.000000	0.500000	0.017 (2)	
Cl1	0.5075 (4)	0.0150 (8)	0.250000	0.0211 (9)	
Cl2	0.8336 (4)	0.6671 (9)	0.4185 (2)	0.0379 (10)	

### Atomic displacement parameters (Å^2^)

**Table:**

	*U*^11^	*U*^22^	*U*^33^	*U*^12^	*U*^13^	*U*^23^
Tl1	0.0378 (7)	0.0378 (7)	0.0369 (8)	0.0189 (3)	0.000	0.000
Tl2	0.0353 (5)	0.0353 (5)	0.0479 (7)	0.0176 (2)	0.000	0.000
Mg1	0.011 (2)	0.011 (2)	0.013 (3)	0.0053 (11)	0.000	0.000
Mg2	0.019 (4)	0.019 (4)	0.014 (5)	0.0094 (19)	0.000	0.000
Cl1	0.0224 (18)	0.011 (2)	0.0261 (19)	0.0055 (10)	0.000	0.000
Cl2	0.0418 (18)	0.027 (2)	0.0399 (18)	0.0135 (10)	−0.0123 (9)	−0.0246 (18)

### Geometric parameters (Å, º)

**Table:**

Tl1—Cl1^i^	3.5126 (2)	Tl2—Cl2^x^	3.5146 (3)
Tl1—Cl1^ii^	3.5126 (2)	Tl2—Cl2^xvi^	3.5146 (3)
Tl1—Cl1^iii^	3.5126 (2)	Tl2—Cl2^xvii^	3.622 (5)
Tl1—Cl1	3.5126 (2)	Tl2—Cl2^xviii^	3.622 (5)
Tl1—Cl1^iv^	3.5126 (2)	Tl2—Cl2^xix^	3.622 (5)
Tl1—Cl1^v^	3.5126 (2)	Mg1—Cl2	2.448 (6)
Tl1—Cl2^vi^	3.576 (5)	Mg1—Cl2^ii^	2.448 (6)
Tl1—Cl2^vii^	3.576 (5)	Mg1—Cl2^vii^	2.448 (6)
Tl1—Cl2^viii^	3.576 (5)	Mg1—Cl1^vii^	2.499 (6)
Tl1—Cl2^ix^	3.576 (5)	Mg1—Cl1	2.499 (6)
Tl1—Cl2^x^	3.576 (5)	Mg1—Cl1^ii^	2.499 (6)
Tl1—Cl2^xi^	3.576 (5)	Mg1—Mg1^xvi^	3.162 (13)
Tl2—Cl1^ii^	3.510 (3)	Mg2—Cl2^xx^	2.476 (4)
Tl2—Cl1^xii^	3.510 (3)	Mg2—Cl2^xxi^	2.476 (4)
Tl2—Cl1^iv^	3.510 (3)	Mg2—Cl2^xxii^	2.476 (4)
Tl2—Cl2^xiii^	3.5146 (3)	Mg2—Cl2^xxiii^	2.476 (4)
Tl2—Cl2^xiv^	3.5146 (3)	Mg2—Cl2^xxiv^	2.476 (4)
Tl2—Cl2^viii^	3.5146 (3)	Mg2—Cl2	2.476 (4)
Tl2—Cl2^xv^	3.5146 (3)		
			
Cl1^i^—Tl1—Cl1^ii^	120.0	Cl1^iv^—Tl2—Cl2^xvi^	123.81 (8)
Cl1^i^—Tl1—Cl1^iii^	57.02 (16)	Cl2^xiii^—Tl2—Cl2^xvi^	175.12 (14)
Cl1^ii^—Tl1—Cl1^iii^	177.02 (16)	Cl2^xiv^—Tl2—Cl2^xvi^	119.820 (11)
Cl1^i^—Tl1—Cl1	62.98 (16)	Cl2^viii^—Tl2—Cl2^xvi^	119.820 (10)
Cl1^ii^—Tl1—Cl1	57.02 (16)	Cl2^xv^—Tl2—Cl2^xvi^	59.85 (17)
Cl1^iii^—Tl1—Cl1	120.0	Cl2^x^—Tl2—Cl2^xvi^	60.03 (17)
Cl1^i^—Tl1—Cl1^iv^	177.02 (16)	Cl1^ii^—Tl2—Cl2^xvii^	176.96 (10)
Cl1^ii^—Tl1—Cl1^iv^	62.98 (16)	Cl1^xii^—Tl2—Cl2^xvii^	119.42 (7)
Cl1^iii^—Tl1—Cl1^iv^	120.0	Cl1^iv^—Tl2—Cl2^xvii^	119.42 (7)
Cl1—Tl1—Cl1^iv^	120.000 (1)	Cl2^xiii^—Tl2—Cl2^xvii^	58.64 (11)
Cl1^i^—Tl1—Cl1^v^	120.000 (1)	Cl2^xiv^—Tl2—Cl2^xvii^	58.64 (11)
Cl1^ii^—Tl1—Cl1^v^	119.999 (1)	Cl2^viii^—Tl2—Cl2^xvii^	88.00 (9)
Cl1^iii^—Tl1—Cl1^v^	62.98 (16)	Cl2^xv^—Tl2—Cl2^xvii^	88.00 (9)
Cl1—Tl1—Cl1^v^	177.02 (16)	Cl2^x^—Tl2—Cl2^xvii^	116.71 (6)
Cl1^iv^—Tl1—Cl1^v^	57.02 (16)	Cl2^xvi^—Tl2—Cl2^xvii^	116.71 (6)
Cl1^i^—Tl1—Cl2^vi^	60.17 (6)	Cl1^ii^—Tl2—Cl2^xviii^	119.42 (7)
Cl1^ii^—Tl1—Cl2^vi^	118.86 (6)	Cl1^xii^—Tl2—Cl2^xviii^	176.96 (10)
Cl1^iii^—Tl1—Cl2^vi^	60.17 (6)	Cl1^iv^—Tl2—Cl2^xviii^	119.42 (7)
Cl1—Tl1—Cl2^vi^	90.84 (4)	Cl2^xiii^—Tl2—Cl2^xviii^	88.00 (9)
Cl1^iv^—Tl1—Cl2^vi^	118.86 (6)	Cl2^xiv^—Tl2—Cl2^xviii^	116.71 (6)
Cl1^v^—Tl1—Cl2^vi^	90.84 (5)	Cl2^viii^—Tl2—Cl2^xviii^	58.64 (11)
Cl1^i^—Tl1—Cl2^vii^	90.84 (4)	Cl2^xv^—Tl2—Cl2^xviii^	116.71 (6)
Cl1^ii^—Tl1—Cl2^vii^	60.17 (6)	Cl2^x^—Tl2—Cl2^xviii^	58.64 (11)
Cl1^iii^—Tl1—Cl2^vii^	118.86 (6)	Cl2^xvi^—Tl2—Cl2^xviii^	88.00 (9)
Cl1—Tl1—Cl2^vii^	60.17 (6)	Cl2^xvii^—Tl2—Cl2^xviii^	58.07 (11)
Cl1^iv^—Tl1—Cl2^vii^	90.84 (4)	Cl1^ii^—Tl2—Cl2^xix^	119.42 (7)
Cl1^v^—Tl1—Cl2^vii^	118.86 (6)	Cl1^xii^—Tl2—Cl2^xix^	119.42 (8)
Cl2^vi^—Tl1—Cl2^vii^	147.12 (6)	Cl1^iv^—Tl2—Cl2^xix^	176.96 (10)
Cl1^i^—Tl1—Cl2^viii^	118.86 (6)	Cl2^xiii^—Tl2—Cl2^xix^	116.71 (6)
Cl1^ii^—Tl1—Cl2^viii^	90.84 (5)	Cl2^xiv^—Tl2—Cl2^xix^	88.00 (9)
Cl1^iii^—Tl1—Cl2^viii^	90.84 (5)	Cl2^viii^—Tl2—Cl2^xix^	116.71 (6)
Cl1—Tl1—Cl2^viii^	118.86 (6)	Cl2^xv^—Tl2—Cl2^xix^	58.64 (11)
Cl1^iv^—Tl1—Cl2^viii^	60.17 (6)	Cl2^x^—Tl2—Cl2^xix^	88.00 (9)
Cl1^v^—Tl1—Cl2^viii^	60.17 (6)	Cl2^xvi^—Tl2—Cl2^xix^	58.64 (11)
Cl2^vi^—Tl1—Cl2^viii^	58.71 (11)	Cl2^xvii^—Tl2—Cl2^xix^	58.07 (11)
Cl2^vii^—Tl1—Cl2^viii^	147.12 (6)	Cl2^xviii^—Tl2—Cl2^xix^	58.07 (11)
Cl1^i^—Tl1—Cl2^ix^	118.86 (6)	Cl2—Mg1—Cl2^ii^	91.8 (2)
Cl1^ii^—Tl1—Cl2^ix^	90.84 (5)	Cl2—Mg1—Cl2^vii^	91.8 (2)
Cl1^iii^—Tl1—Cl2^ix^	90.84 (5)	Cl2^ii^—Mg1—Cl2^vii^	91.8 (2)
Cl1—Tl1—Cl2^ix^	118.86 (6)	Cl2—Mg1—Cl1^vii^	91.84 (10)
Cl1^iv^—Tl1—Cl2^ix^	60.17 (6)	Cl2^ii^—Mg1—Cl1^vii^	91.84 (10)
Cl1^v^—Tl1—Cl2^ix^	60.17 (6)	Cl2^vii^—Mg1—Cl1^vii^	174.7 (3)
Cl2^vi^—Tl1—Cl2^ix^	147.12 (6)	Cl2—Mg1—Cl1	174.7 (3)
Cl2^vii^—Tl1—Cl2^ix^	58.71 (11)	Cl2^ii^—Mg1—Cl1	91.84 (10)
Cl2^viii^—Tl1—Cl2^ix^	111.04 (14)	Cl2^vii^—Mg1—Cl1	91.84 (10)
Cl1^i^—Tl1—Cl2^x^	90.84 (4)	Cl1^vii^—Mg1—Cl1	84.3 (2)
Cl1^ii^—Tl1—Cl2^x^	60.17 (6)	Cl2—Mg1—Cl1^ii^	91.84 (10)
Cl1^iii^—Tl1—Cl2^x^	118.86 (6)	Cl2^ii^—Mg1—Cl1^ii^	174.7 (3)
Cl1—Tl1—Cl2^x^	60.17 (6)	Cl2^vii^—Mg1—Cl1^ii^	91.84 (10)
Cl1^iv^—Tl1—Cl2^x^	90.84 (5)	Cl1^vii^—Mg1—Cl1^ii^	84.3 (2)
Cl1^v^—Tl1—Cl2^x^	118.86 (6)	Cl1—Mg1—Cl1^ii^	84.3 (2)
Cl2^vi^—Tl1—Cl2^x^	58.71 (11)	Cl2^xx^—Mg2—Cl2^xxi^	90.17 (16)
Cl2^vii^—Tl1—Cl2^x^	111.04 (14)	Cl2^xx^—Mg2—Cl2^xxii^	89.83 (16)
Cl2^viii^—Tl1—Cl2^x^	58.71 (11)	Cl2^xxi^—Mg2—Cl2^xxii^	180.0
Cl2^ix^—Tl1—Cl2^x^	147.12 (6)	Cl2^xx^—Mg2—Cl2^xxiii^	90.17 (16)
Cl1^i^—Tl1—Cl2^xi^	60.17 (6)	Cl2^xxi^—Mg2—Cl2^xxiii^	90.17 (17)
Cl1^ii^—Tl1—Cl2^xi^	118.86 (6)	Cl2^xxii^—Mg2—Cl2^xxiii^	89.83 (17)
Cl1^iii^—Tl1—Cl2^xi^	60.17 (6)	Cl2^xx^—Mg2—Cl2^xxiv^	89.83 (16)
Cl1—Tl1—Cl2^xi^	90.84 (4)	Cl2^xxi^—Mg2—Cl2^xxiv^	89.83 (17)
Cl1^iv^—Tl1—Cl2^xi^	118.86 (6)	Cl2^xxii^—Mg2—Cl2^xxiv^	90.17 (17)
Cl1^v^—Tl1—Cl2^xi^	90.84 (5)	Cl2^xxiii^—Mg2—Cl2^xxiv^	180.0
Cl2^vi^—Tl1—Cl2^xi^	111.04 (14)	Cl2^xx^—Mg2—Cl2	180.0
Cl2^vii^—Tl1—Cl2^xi^	58.71 (11)	Cl2^xxi^—Mg2—Cl2	89.83 (17)
Cl2^viii^—Tl1—Cl2^xi^	147.12 (6)	Cl2^xxii^—Mg2—Cl2	90.17 (17)
Cl2^ix^—Tl1—Cl2^xi^	58.71 (11)	Cl2^xxiii^—Mg2—Cl2	89.83 (16)
Cl2^x^—Tl1—Cl2^xi^	147.12 (6)	Cl2^xxiv^—Mg2—Cl2	90.17 (16)
Cl1^ii^—Tl2—Cl1^xii^	63.03 (10)	Mg1^xvi^—Cl1—Mg1	78.5 (3)
Cl1^ii^—Tl2—Cl1^iv^	63.03 (10)	Mg1^xvi^—Cl1—Tl2^xxv^	87.89 (12)
Cl1^xii^—Tl2—Cl1^iv^	63.03 (10)	Mg1—Cl1—Tl2^xxv^	166.36 (18)
Cl1^ii^—Tl2—Cl2^xiii^	123.81 (8)	Mg1^xvi^—Cl1—Tl2^xxvi^	166.36 (18)
Cl1^xii^—Tl2—Cl2^xiii^	91.92 (9)	Mg1—Cl1—Tl2^xxvi^	87.89 (12)
Cl1^iv^—Tl2—Cl2^xiii^	60.79 (8)	Tl2^xxv^—Cl1—Tl2^xxvi^	105.74 (13)
Cl1^ii^—Tl2—Cl2^xiv^	123.81 (8)	Mg1^xvi^—Cl1—Tl1^xxvii^	91.16 (6)
Cl1^xii^—Tl2—Cl2^xiv^	60.79 (8)	Mg1—Cl1—Tl1^xxvii^	91.15 (6)
Cl1^iv^—Tl2—Cl2^xiv^	91.92 (9)	Tl2^xxv^—Cl1—Tl1^xxvii^	89.10 (5)
Cl2^xiii^—Tl2—Cl2^xiv^	59.85 (17)	Tl2^xxvi^—Cl1—Tl1^xxvii^	89.10 (5)
Cl1^ii^—Tl2—Cl2^viii^	91.92 (9)	Mg1^xvi^—Cl1—Tl1	91.15 (6)
Cl1^xii^—Tl2—Cl2^viii^	123.81 (8)	Mg1—Cl1—Tl1	91.15 (6)
Cl1^iv^—Tl2—Cl2^viii^	60.79 (8)	Tl2^xxv^—Cl1—Tl1	89.10 (5)
Cl2^xiii^—Tl2—Cl2^viii^	60.03 (17)	Tl2^xxvi^—Cl1—Tl1	89.10 (5)
Cl2^xiv^—Tl2—Cl2^viii^	119.820 (10)	Tl1^xxvii^—Cl1—Tl1	177.02 (16)
Cl1^ii^—Tl2—Cl2^xv^	91.92 (9)	Mg1—Cl2—Mg2	178.8 (3)
Cl1^xii^—Tl2—Cl2^xv^	60.79 (8)	Mg1—Cl2—Tl2^xvi^	88.60 (7)
Cl1^iv^—Tl2—Cl2^xv^	123.81 (8)	Mg2—Cl2—Tl2^xvi^	91.44 (7)
Cl2^xiii^—Tl2—Cl2^xv^	119.820 (10)	Mg1—Cl2—Tl2^xxviii^	88.60 (7)
Cl2^xiv^—Tl2—Cl2^xv^	60.03 (17)	Mg2—Cl2—Tl2^xxviii^	91.44 (7)
Cl2^viii^—Tl2—Cl2^xv^	175.12 (14)	Tl2^xvi^—Cl2—Tl2^xxviii^	175.12 (14)
Cl1^ii^—Tl2—Cl2^x^	60.79 (8)	Mg1—Cl2—Tl1^xxix^	90.52 (17)
Cl1^xii^—Tl2—Cl2^x^	123.81 (8)	Mg2—Cl2—Tl1^xxix^	90.67 (16)
Cl1^iv^—Tl2—Cl2^x^	91.92 (9)	Tl2^xvi^—Cl2—Tl1^xxix^	88.02 (8)
Cl2^xiii^—Tl2—Cl2^x^	119.820 (11)	Tl2^xxviii^—Cl2—Tl1^xxix^	88.02 (8)
Cl2^xiv^—Tl2—Cl2^x^	175.12 (14)	Mg1—Cl2—Tl2^xxx^	89.9 (2)
Cl2^viii^—Tl2—Cl2^x^	59.85 (17)	Mg2—Cl2—Tl2^xxx^	88.94 (11)
Cl2^xv^—Tl2—Cl2^x^	119.820 (11)	Tl2^xvi^—Cl2—Tl2^xxx^	91.99 (9)
Cl1^ii^—Tl2—Cl2^xvi^	60.79 (8)	Tl2^xxviii^—Cl2—Tl2^xxx^	91.99 (9)
Cl1^xii^—Tl2—Cl2^xvi^	91.92 (9)	Tl1^xxix^—Cl2—Tl2^xxx^	179.61 (13)

Symmetry codes: (i) −*y*, *x*−*y*−1, *z*; (ii) −*x*+*y*+1, −*x*+1, *z*; (iii) −*x*+*y*, −*x*, *z*; (iv) −*y*, *x*−*y*, *z*; (v) *x*−1, *y*, *z*; (vi) *x*−1, *y*−1, −*z*+1/2; (vii) −*y*+1, *x*−*y*, *z*; (viii) −*x*+*y*, −*x*+1, −*z*+1/2; (ix) −*x*+*y*, −*x*+1, *z*; (x) −*y*+1, *x*−*y*, −*z*+1/2; (xi) *x*−1, *y*−1, *z*; (xii) *x*, *y*+1, *z*; (xiii) *x*−1, *y*, −*z*+1/2; (xiv) −*y*+1, *x*−*y*+1, −*z*+1/2; (xv) −*x*+*y*+1, −*x*+2, −*z*+1/2; (xvi) *x*, *y*, −*z*+1/2; (xvii) *x*−*y*, *x*, *z*−1/2; (xviii) −*x*+1, −*y*+1, *z*−1/2; (xix) *y*, −*x*+*y*+1, *z*−1/2; (xx) −*x*+2, −*y*+2, −*z*+1; (xxi) *x*−*y*+1, *x*, −*z*+1; (xxii) −*x*+*y*+1, −*x*+2, *z*; (xxiii) *y*, −*x*+*y*+1, −*z*+1; (xxiv) −*y*+2, *x*−*y*+1, *z*; (xxv) *x*, *y*−1, *z*; (xxvi) *x*, *y*−1, −*z*+1/2; (xxvii) *x*+1, *y*, *z*; (xxviii) *x*+1, *y*, −*z*+1/2; (xxix) *x*+1, *y*+1, *z*; (xxx) −*x*+1, −*y*+1, *z*+1/2.
