# Supplementary material for: The crystal structure of TlMgCl3 from 290 K to 725 K
Source: Acta Crystallogr E Crystallogr Commun. 2020 Oct 6;76(Pt 11):1716–9. doi: 10.1107/S2056989020013201 (PMC7643245; doi:10.1107/S2056989020013201)
Supplement: Supplementary file 1 [file e-76-01716-sup3.odt]

Supporting information

The Crystal Structure of TlMgCl3 from 290 K to 725 K

Drew R. Onken, Didier Perrodin, Sven C. Vogel, Edith D. Bourret, & Federico Moretti      

For the determination of the effective atomic number, Zeff, the authors used the empirical formula from Derenzo & Choong (2009): 

where for element i, ci is the count in the chemical formula, wi  is the atomic number, and Zi is the atomic number. Derenzo & Choong (2009) determined that x0 = 3 is best. This equation yields Zeff = 67 for TlMgCl3.

Supplemental Figure 1 shows the powder neutron diffraction (ND) curves for increasing and decreasing temperatures. Supplemental Figure 2 shows a sample Rietveld fit for the 90° detector bank, applying the crystal structure measured by SC-XRD in this work to the ND data at 300 K. Supplemental Table 1 gives the weighted pattern R index (Rwp) and the fit error (𝜒2) for each temperature. Supplemental Table 2 compares the refined structure parameters from the ND data at 300 K to the structure reported in this work from single-crystal X-ray diffraction (SC-XRD) at 290 K.

Supplemental Figure 1: High-temperature neutron diffraction shows the stability of the TlMgCl3 phase as the sample is heated from 300 K to 725 K and then cooled back down to 399 K. There is no apparent hysteresis in the diffraction patterns.

Supplemental Figure 2: The hexagonal crystal structure determined by single-crystal X-ray diffraction fits the power neutron diffraction data. This example is for data collected by the 90° detector ring at 425 K and is representative of the series. The experimental data is indicated by the red + symbols; the green line indicates the Rietveld fit; and the pink line below indicates the difference between these. The small black ticks mark peak positions.

Supplemental Table 1: Overview of the temperatures at which ND data were collected, and the accuracy of the Rietveld fit to the experimental data.

| Temperature [K] | Heating/Cooling | Rwp | 𝜒2 |
| --- | --- | --- | --- |
| 300 | Heating | 0.0116 | 5.78 |
| 350 | Heating | 0.0086 | 2.36 |
| 425 | Heating | 0.0085 | 2.99 |
| 537 | Heating | 0.0085 | 2.02 |
| 607 | Heating | 0.0084 | 2.25 |
| 636 | Heating | 0.0081 | 2.81 |
| 725 | Heating | 0.0078 | 2.60 |
| 680 | Cooling | 0.0083 | 2.18 |
| 622 | Cooling | 0.0086 | 2.28 |
| 586 | Cooling | 0.0086 | 2.31 |
| 483 | Cooling | 0.0087 | 3.09 |
| 399 | Cooling | 0.0094 | 2.55 |

Supplemental Table 2: Crystal structure parameters (isotropic thermal displacement UISO and atomic positions X,Y,Z) are compared for refinements on two different diffraction measurements: from SC-XRD at 290 K from ND at 300 K. The SC-XRD measurements are used for the crystal structure cif file enclosed with this work. The ND data used that structure as starting values for Rietveld refinement. Compared to the parameters from SC-XRD, the parameters from ND are similar, but the difference is statistically significant based on the reported errors from the Rietveld fit (this may not include all systematic errors for each instrument). *For ND data at 300 K, the lattice parameters were fixed from the SC-XRD values, extrapolated up 10 degrees to 300 K using the coefficients of thermal expansion from a previous iteration of the Rietveld analysis. The fixed lattice parameters were used at 300 K so that proper instrument calibration parameters could be determined for the rest of the ND data series.

|  | SC-XRD at 290 K | ND at 300 K |
| --- | --- | --- |
| a | 7.0228(4) | 7.0250* |
| c | 17.4934(15) | 17.4972* |
| Vol | 747.18(11) | 747.811* |
| Tl1 UISO | 0.0375(5) | 0.046(3) |
| Tl2 UISO | 0.0395(4) | 0.023(1) |
| Mg1 UISO | 0.0115(14) | 0.011(2) |
| Mg2 UISO | 0.017(2) | 0.015(2) |
| Cl1 UISO | 0.0211(9) | 0.0212(6) |
| Cl2 UISO | 0.0379(10) | 0.0358(8) |
| Tl2 Z | 0.09002(7) | 0.0938(2) |
| Mg1 Z | 0.3404(4) | 0.3412(3) |
| Cl1 X | 0.5075(4) | 0.5065(5) |
| Cl1 Y | 0.0150(8) | 0.0129(9) |
| Cl2 X | 0.8336(4) | 0.8298(4) |
| Cl2 Y | 0.6671(9) | 0.6596(7) |
| Cl2 Z | 0.4185(2) | 0.4202(1) |
